# Inter-specific connectivity and stability of the unique relic plant *Chunia bucklandioides* in a community at different structural levels on Hainan Island, China

**DOI:** 10.3389/fpls.2026.1773354

**Published:** 2026-03-30

**Authors:** Shunwei Zhang, Xin Su, Ling Dai, Xiaobo Yang, Dongyang Chen, Dongling Qi, Shaocui He, Naiyan Shang, Lei Yu, Wanqing Wang, Binbin Jiang

**Affiliations:** 1 School of Ecology, Hainan University, Haikou, China; 2 Rubber Research Institute, Sanya Research Institute, China Academy of Tropical Agricultural Sciences, Haikou, China; 3 Hainan Xinlüshen Tropical Bio-engineering Co., Ltd., Haikou, China

**Keywords:** *Chunia bucklandioides*, community stability, endangered plant, interspecific association, relict plant

## Abstract

**Introduction:**

Relic plants are crucial components of biodiversity. Studying the interspecific relationships and stability of endangered relic plants at various structural levels within communities helps to understand their survival status and provides a scientific foundation for conserving unique germplasm resources and biodiversity.

**Methods:**

This study aims to address the following scientific questions: How does species composition across different distribution areas influence interspecific relationships? Which plant species impact the survival of *Chunia bucklandioides* populations in these areas? Are there differences in community stability at various structural levels? Based on survey data from the mountainous communities of *Chunia bucklandioides* in the Jianfengling and Di’anshan sections of Hainan Tropical Rainforest National Park, this study uses the variance ratio method (VR), test statistics (W), chi-squared test (χ^2^), AC connectivity coefficient, percentage co-occurrence (PC) test, and an improved stability analysis method developed by M. Godron to analyze interspecific relationships and community stability at different structural levels.

**Results:**

The results show that: (1) The communities inhabited by *Chunia bucklandioides* have rich species composition, and there are significant differences in community structure between the Jianfengling area and the Diaoluoshan area. (2) In both distribution regions, positive interspecific associations dominate among species in different forest layers of the *Chunia bucklandioides* communities, with strong interspecific association in the arbor layer and weak interspecific association in the shrub layer and herb layer. (3) Different structural layers of the *Chunia bucklandioides* communities in both the Jianfengling area and the Diaoluoshan area are in an unstable state. In terms of stability, the performance of the two distribution regions is as follows: Diaoluoshan area community > Jianfengling area community; the order is reversed among different forest layers—for the Jianfengling area: arbor layer > shrub layer > herb layer; for the Diaoluoshan area: herb layer > shrub layer > arbor layer. (4) Mature individuals of *Chunia bucklandioides* occupy an absolute dominant position in the community, while juvenile individuals are under highly competitive pressure, leading to restricted population regeneration.

**Discussion:**

This research provides valuable insights into the interspecific relationships, community stability, and succession dynamics of endangered *Chunia bucklandioides* populations, offering key scientific knowledge for their conservation and practical guidance for ecosystem restoration in tropical rainforests.

## 1 Introduction

The current biodiversity pattern results from long-term co-selection by ecological environments throughout history. Among these, relict plants have survived significant environmental changes over millions of years, serving as important indicators for studying both past and recent biogeographic and evolutionary processes. Compared to non-relict populations, relict plants are more vulnerable to severe threats from human activities, and their extinction risk is steadily increasing, posing a significant challenge to global biodiversity (Tang et al., 2023, Kozlowski and Gratzfeld, 2013). Hainan Island in China is home to the largest contiguous area of tropical rainforest in the country, with extremely high biodiversity, serving as a refuge for many relict and rare plant species (Zong, 2020, Tian et al., 2018). However, industrial logging in the mid-20th century, the introduction of rubber plantations, and the recent expansion of areca nut cultivation have caused a gradual decline in the island’s tropical rainforests. Lowland rainforests at relatively low elevations, with abundant water and heat, have experienced the most severe disturbances (Lin et al., 2017), leading to a sharp reduction in the population sizes of relict plants, with some species already endangered (Tang et al., 2024). The extinction of any rare relict plant species represents an irreversible loss to global biodiversity. Therefore, understanding the current survival status of relict and endangered plants is crucial for the conservation of endemic germplasm resources and biodiversity.

A plant community is an assemblage formed by interactions among different plant populations and their long-term adaptation to environmental changes (Zhang et al., 2022). As an important indicator of plant communities, interspecific associations are often analyzed alongside other ecological parameters to study community characteristics (Ding et al., 2023). The combined analysis of interspecific association and niche has been applied in natural plant communities, plantation communities, and endangered plant communities (Hu et al., 2022, Xiao et al., 2024, Gao et al., 2025), providing a scientific basis for understanding plant community competition mechanisms, managing plantations, and conserving endangered species. In recent years, integrating studies on interspecific association and community stability has become a prominent focus in ecological research, particularly in communities with endangered plants. By analyzing the interspecific relationships among dominant tree species and community stability in endangered plant communities, researchers can assess the successional stage of the community (Xie et al., 2021); (Zhang et al., 2019), revealing aspects such as the community’s status (Li et al., 2025, Wei et al., 2022) and distribution trends (Ruan et al., 2024). Species allocation across vertical structural layers of tropical rainforests recovering from disturbances is not evident (Poorter et al., 2021). Species composition and interspecific association of each layer are the primary manifestations reflecting the community succession process, as well as the core drivers maintaining the overall disturbance resistance and stability of the community, which can directly determine the survival adaptability of relict and endangered plant species (Feng et al., 2022, He et al., 2025). Therefore, in tropical rainforests, investigating the interspecific association and stability of the arbor, shrub, and herb layers in communities where relict and endangered plants occur can compensate for the neglect of interlayer microhabitat interactions in community studies at the whole-stand scale. This research can facilitate a better understanding of the resource utilization relationships among species in relict and endangered plant communities and the survival status of endangered populations, thus bearing positive significance for the conservation of relict plant species.

*Chunia bucklandioides* (genus *Chunia*, family Hamamelidaceae) is a monotypic species endemic to Hainan Island, China (Meng et al., 2016; Van et al., 2020). It has a narrow distribution, with rare wild populations found only in the valley rainforests of the Jianfengling and Diaoluoshan areas of Hainan Tropical Rainforest National Park (Zhou et al., 2019). Due to its highly restricted geographical distribution, population recovery has been slow, and the species was listed as a national second-class protected species in the 2021 edition of the National Key Protected Wild Plants List (Zhang et al., 2024). Research on *Chunia bucklandioides* has primarily focused on its genetic characteristics (Zhou et al., 2019) and population traits (Gui et al., 2024), while studies on its associated communities have yet to be reported. Therefore, based on field survey data from *Chunia bucklandioides* habitats, this study investigates the species composition, interspecific associations, and community stability of its associated communities. The study aims to address the following scientific questions: (1) How does species composition in different distribution areas affect interspecific relationships? (2) Which plant species influence the survival of *Chunia bucklandioides* populations in these areas? (3) Are there differences in community stability across different structural layers?

## 2 Materials and methods

### 2.1 Study area

**Figure 1:**
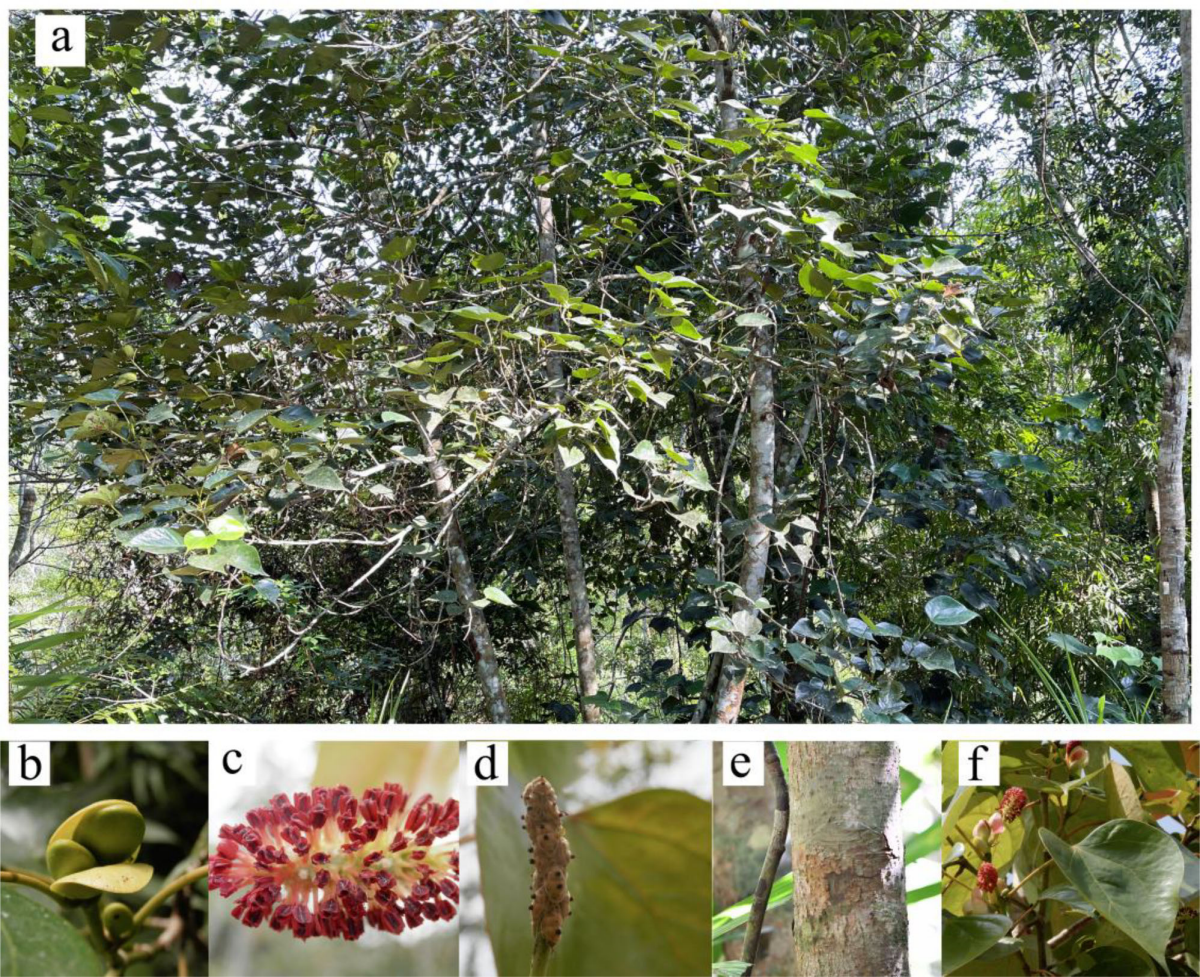
*Chunia bucklandioides* plant. **(a)** *Chunia bucklandioides*; **(b)** bud; **(c)** flower; **(d)** fruit; **(e)** trunk and **(f)** leaves.

**Figure 2:**
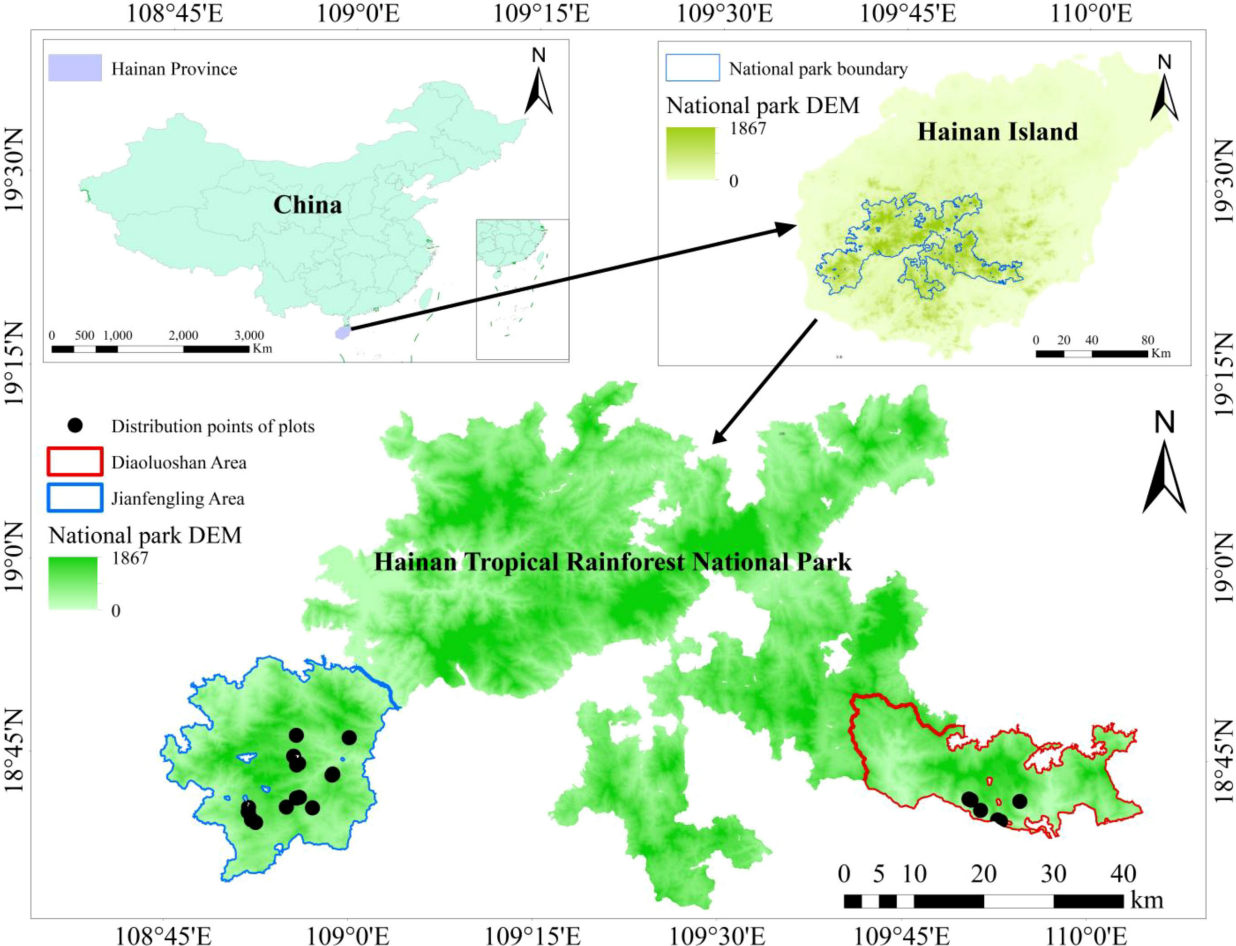
Sampling points for Chunia bucklandioides survey in Hainan Island, China.

Reconnaissance surveys were conducted in the potential distribution area of *Chunia bucklandioides* (**Figure 1**), with the study area shown in **Figure 2**. Natural populations of *Chunia bucklandioides* were found only in the Jianfengling and Diaoluoshan areas of Hainan Tropical Rainforest National Park. The Jianfengling area is located in the southwestern part of Hainan Province (108°36′E–109°05′E, 18°23′N–18°52′N), primarily within Ledong Li Autonomous County, with a small portion in Dongfang City, covering a total area of 20,170 hectares. The Diaoluoshan area is located at the borders of Lingshui, Baoting, and other counties in Hainan Province (109°45′E–110°03′E, 18°43′N–18°58′N). It lies at the same latitude as Jianfengling, forming a geographically corresponding pair. Both areas are within the tropical marine monsoon climate zone, characterized by abundant water and heat, and they contain some of the largest and best-preserved tropical rainforests in China.

### 2.2 Plot setup and survey methodology

Populations of *Chunia bucklandioides* are distributed in valley rainforests with a sparse individual density. For the populations of *Chunia bucklandioides* recorded during reconnaissance surveys, 20 m × 20 m sampling plots were established to conduct community investigations. In the Jianfengling area, where the terrain is relatively gentle and the disturbance intensity is high, a total of 20 sampling plots were recorded, covering a total area of 0.8 hm^2^; in the Diaoluoshan area, characterized by a steep and rugged terrain and low disturbance intensity, six sampling plots were surveyed, with a total area of 0.24 hm^2^. In addition, each plot was divided into four 10 m × 10 m subplots for arbor surveys, where all woody plants with a height (H) ≥ 3 m were measured for diameter at breast height (DBH), tree height, and crown width. Using the five-point sampling method, one 5 m × 5 m shrub subplot was established at each of the four corners and the center of the main plot. In these shrub subplots, all woody plants with 0.5 m ≤ H < 3 m were recorded, measuring DBH or basal diameter, height, and crown width or coverage. For herbaceous plants, one 1 m × 1 m subplot was randomly established within each of the four 10 m × 10 m tree subplots. In these herb subplots, all herbaceous plants and tree seedlings with H < 0.5 m were recorded, noting the number of individuals, height, and coverage.

### 2.3 Importance value

The Importance Value (IV) was used to evaluate the dominant degree of plant species in the community (Whittaker, 1965), which was synthesized by three quantitative indices: Relative Abundance (RA), Relative Frequency (RF), and Relative Coverage (RC). The calculation definitions and formulas of each index and the comprehensive IV are as follows:

(1) $$\begin{array}{c} \text{Relative}\;\text{Abundance}(RA)=(\frac{N_{i}}{N_{total}})\times 100\% \end{array}$$

(2) $$\begin{array}{c} \text{Relative}\;\text{Frequency}\;(RF)=(\frac{F_{i}}{F_{total}})\times 100\% \end{array}$$

(3) $$\begin{array}{c} \text{Relative}\;\text{Dominance}(RD)=(\frac{D_{i}}{D_{total}})\times 100\% \end{array}$$

(4) $$\begin{array}{c} \text{Relative}\;\text{Coverage}\;(RC)=(\frac{C_{i}}{C_{total}})\times 100\% \end{array}$$

(5) $$\begin{array}{c} IV_{ar}=\frac{RA+RF+RD}{3} \end{array}$$

(6) $$\begin{array}{c} IV_{sh}/IV_{he}=\frac{RA+RF+RC}{3} \end{array}$$

### 2.4 Principal coordinates analysis

Principal Coordinates Analysis (PCoA) was conducted in the vegan package of R software to examine the differences in species compositional dissimilarity among different distribution regions (Anderson and Willis, 2003).

### 2.5 Interspecific association

The Variance Ratio (VR) method (Schluter, 1984) was used to determine the overall association among multiple species, with the test statistic W employed to assess the significance of the association. A VR > 1 indicates a positive interspecific association, VR < 1 indicates a negative association, and VR = 1 indicates no association.

(7) $$\begin{array}{c} VR=\frac{\frac{1}{n}\sum_{j=1}^{n}\;{\left(T_{j}-t\right)}^{2}}{\sum_{j=1}^{S}(1-\frac{n_{i}}{n})\frac{n_{i}}{n}} \end{array}$$

(8) $$\begin{array}{c} W=VR\cdot N \end{array}$$

Where:

S is the total number of populations studied.
N is the total number of quadrats.
n_i_ is the number of quadrats in which population i appears.
T_j_ is the number of populations present in quadrat j.
t is the average number of populations per quadrat, calculated as.
t = (t_1_+t_2_+…+t_n_)/N.

The Yates-corrected chi-square (χ2) test (Huang et al., 2017) was used to qualitatively determine the association between each pair of species. A χ^2^ value < 3.841 indicates no significant association, while χ^2^ > 3.841 *indicates* a significant association.

(9) $$\begin{array}{c} \chi^{2}\;=\frac{N{\left(|ad-bc|-0.5N\right)}^{2}}{\;(a+b)(a+c)(c+d)(b+d)} \end{array}$$

Where:

N is the total number of quadrats;
a is the number of quadrats in which both plant species occur.
b and c are the number of quadrats in which only one of the two plant species occur.
d is the number of quadrats in which neither of the two plant species occurs.

The Association Coefficient (AC) and Percentage of Co-occurrence (PC) tests (Su et al., 2007) were employed. The AC value ranges from [-1, 1], where values closer to 1 indicate a stronger positive association, values closer to -1 indicate a stronger negative association, and a value of 0 indicates no association. The PC value ranges from [0, 1], with values closer to 1 indicating a stronger positive association and values closer to 0 indicating weaker association.

(10) $$\begin{array}{c} ad\;\geq \;bc,\;AC\;=\;\frac{ad-bc}{\left(a+b)(b+d\right)} \end{array}$$

(11) $$\begin{array}{c} ad\;<\;bc,\;d\;\geq \;a,\;AC\;=\;\frac{ad-bc}{\left(a+b)(a+c\right)} \end{array}$$

(12) $$\begin{array}{c} ad\;<\;bc,\;d\;<\;a,\;AC\;=\;\frac{ad-bc}{\left(b+d)(c+d\right)} \end{array}$$

(13) $$\begin{array}{c} PC\;=\;\frac{a}{a+b+c} \end{array}$$

Where:

N is the total number of quadrats.
a is the number of quadrats in which both species A and B occur.
b is the number of quadrats in which only species A occurs.
c is the number of quadrats in which only species B occurs.
d is the number of quadrats in which neither species A nor B occurs.

### 2.6 Community stability

Community stability was analyzed using the modified M. Godron stability method (Zheng, 2000). This method plots the cumulative percentage of total plant individuals on the x-axis and cumulative relative frequency on the y-axis, fitting a smooth curve to the data (quadratic regression model: y = ax^2^ + bx + c). The stability of the community is determined by the proximity of the intersection point between this curve and the line y = -x + 100 to the theoretical stability point (20, 80). Communities with intersection points closer to (20, 80) are considered more stable.

(14) $$\begin{array}{c} \rho =\sqrt{{\left(x_{2}-x_{1}\right)}^{2}+{\left(y_{2}-y_{1}\right)}^{2}} \end{array}$$

Where:

*ρ* is the Euclidean distance.
(x1, y1) are the coordinates of the stability point (20, 80).
(x2, y2) are the coordinates of the intersection point between the M. Godron stability equation and the line y = -x + 100.

### 2.7 Data analysis

Microsoft Excel 2021 was used for data processing and creating charts related to interspecific associations. The chi-square (χ^2^) test was performed using the spaa package in R 4.4.2. ArcGIS 10.8 was employed to generate the study area location map. Origin 2025 was used for fitting the M. Godron scatter plot curves.

## 3 Results

### 3.1 Species composition and community structure of *Chunia bucklandioides* habitats

In the Jianfengling area, a total of 5,230 vascular plant individuals were recorded in the communities containing *Chunia bucklandioides*, belonging to 85 families, 199 genera, and 354 species. In the Diaoluoshan area, 1,553 vascular plant individuals were recorded, belonging to 69 families, 164 genera, and 262 species (**Table 1**). Overall, species in the communities of both sections were classified into three major plant groups, with angiosperms being the dominant group. The Jianfengling community had a higher number of fern and angiosperm species compared to the Diaoluoshan community, while the number of gymnosperm species was identical between the two areas.

**Table 1:** Species composition of *Chunia bucklandioides* community.

Distribution area	Plant group	Family	Family of sections	Genus	Genus of sections	Species	Species of sections
Jianfengling Area	pteridophyte	12	14.12%	14	7.04%	18	5.08%
	gymnosperm	1	1.18%	1	0.50%	1	0.28%
	angiosperm	72	84.71%	184	92.46%	335	94.63%
	total	85	100.00%	199	100.00%	354	100.00%
Diaoluoshan Area	pteridophyte	7	10.14%	7	4.27%	7	2.67%
	gymnosperm	1	1.45%	1	0.61%	1	0.38%
	angiosperm	61	88.41%	156	95.12%	254	96.95%
	total	69	100.00%	164	100.00%	262	100.00%

In all communities where *Chunia bucklandioides* occurs, it is the dominant species, with high abundance and absolute dominance in the arbor, shrub, and herb layers. Its importance value (Equations 1–6) is significantly greater than that of any other species (**Table 2**). Excluding *Chunia bucklandioides*, the top ten species in terms of importance value vary somewhat between the two study sites.

**Table 2:** Importance values of dominant arbor species in different forest layers of the community with *Chunia bucklandioides*.

Distribution area	Forest layer	Ranking	Species	Abbreviation	Importance value
Jianfengling Area	Arbor layer	1	*Chunia bucklandioides*	Chu	0.4868
		2	*Gironniera subaequalis*	Gir	0.3332
		3	*Ormosia balansae*	Orm	0.2813
		4	*Garcinia oblongifolia*	Gar	0.2726
		5	*Canarium album*	Can	0.2646
		6	*Engelhardia roxburghiana*	Eng	0.2493
		7	*Carallia brachiata*	Car	0.2409
		8	*Adinandra hainanensis*	Adi	0.2392
		9	*Castanopsis jianfenglingensis*	Cas	0.2307
		10	*Schima superba*	Sch	0.2273
	Shrub layer	1	*Chunia bucklandioides*	Chu	0.4152
		2	*Gironniera subaequalis*	Gir	0.2839
		3	*Psychotria asiatica*	Psy	0.2180
		4	*Gomphandra tetrandra*	Gom	0.2107
		5	*Tabernaemontana bufalina*	Tab	0.1942
		6	*Beilschmiedia laevis*	Bei	0.1918
		7	*Glochidion coccineum*	Glo	0.1913
		8	*Prismatomeris tetrandra*	Pri	0.1779
		9	*Lasianthus trichophlebus* var. *latifolius*	Las	0.1773
		10	*Ficus vasculosa*	Fic	0.1771
	Herb layer	1	*Chunia bucklandioides*	Chu	1.2574
		2	*Licuala hainanensis*	Lic	0.2233
		3	*Calamus tetradactylus*	Cal	0.2003
		4	*Pandanus austrosinensis*	Pan	0.1747
		5	*Lygodium longifolium*	Lyg	0.1584
		6	*Licuala fordiana*	Licu	0.1576
		7	*Madhuca hainanensis*	Mad	0.1540
		8	*Garcinia oblongifolia*	Gar	0.1530
		9	*Dinochloa orenuda*	Din	0.1525
		10	*Castanopsis fissa*	Cas	0.1489
Diaoluoshan Area	Arbor layer	1	*Chunia bucklandioides*	Chu	0.4357
		2	*Alphonsea monogyna*	Alph	0.3535
		3	*Vatica mangachapoi*	Vat	0.3432
		4	*Ficus vasculosa*	Fic	0.3062
		5	*Gironniera subaequalis*	Gir	0.2996
		6	*Maclurodendron oligophlebium*	Mac	0.2968
		7	*Schefflera heptaphylla*	Sch	0.2954
		8	*Garcinia oblongifolia*	Gar	0.2901
		9	*Nephelium topengii*	Nep	0.2900
		10	*Sarcosperma laurinum*	Sar	0.2891
	Shrub layer	1	*Chunia bucklandioides*	Chu	0.4544
		2	*Ardisia crenata*	Ard	0.3050
		3	*Aidia canthioides*	Aid	0.2930
		4	*Psychotria asiatica*	Psy	0.2531
		5	*Saprosma merrillii*	Sap	0.2421
		6	*Ficus vasculosa*	Fic	0.2397
		7	*Garcinia oblongifolia*	Gar	0.2384
		8	*Symplocos ovatilobata*	Sym	0.2363
		9	*Meliosma angustifolia*	Mel	0.2327
		10	*Gomphandra tetrandra*	Gom	0.2325
	Herb layer	1	*Chunia bucklandioides*	Chu	0.6181
		2	*Schizostachyum hainanense*	Sch	0.4130
		3	*Pronephrium simplex*	Pro	0.2615
		4	*Alpinia coriacea*	Alp	0.2355
		5	*Hedyotis paridifolia*	Hed	0.2131
		6	*Sterculia hainanensis*	Ste	0.1938
		7	*Dichapetalum gelonioides*	Dic	0.1902
		8	*Sterculia lanceolata*	Ste	0.1513
		9	*Alphonsea monogyna*	Alph	0.1446
		10	*Scleria terrestris*	Scl	0.1430

In the arbor layer, *Gironniera subaequalis* Planch. and *Garcinia oblongifolia* Champ. ex Benth. are common dominant species in both areas. Other dominant arbor species differ between the sites: Jianfengling contains *Ormosia balansae* Drake, *Canarium album* (Lour.) Raeusch. ex DC., and *Castanopsis jianfenglingensis* Duanmu, while Diaoluoshan includes *Alphonsea monogyna* Merr. & Chun, *Vatica mangachapoi* Blanco, and *Maclurodendron oligophlebium* (Merrill) T. G. Hartley.

In the shrub layer, *Psychotria asiatica* L., *Gomphandra tetrandra* (Wall.) Sleumer., and *Ficus vasculosa* Wall. ex Miq. are co-dominant species. Additionally, the Jianfengling community also includes *Gironniera subaequalis* Planch., *Tabernaemontana bufalina* Lour., and *Prismatomeris tetrandra* (Roxb.) K. Schum., while the Diaoluoshan community includes *Ardisia crenata* Sims and *Aidia canthioides* (Champ. ex Benth.) Masam.

There are significant differences in the dominant species of the herb layer between the two regions: the Jianfengling community contains species such as *Licuala hainanensis* A. J. Hend., *L. X. Guo* & Barfod, and *Licuala fordiana* Becc., while the Diaoluoshan community includes *Schizostachyum hainanense* Merr. ex McClure and *Pronephrium simplex* (Hook.) Holttum.

**Figure 3:**
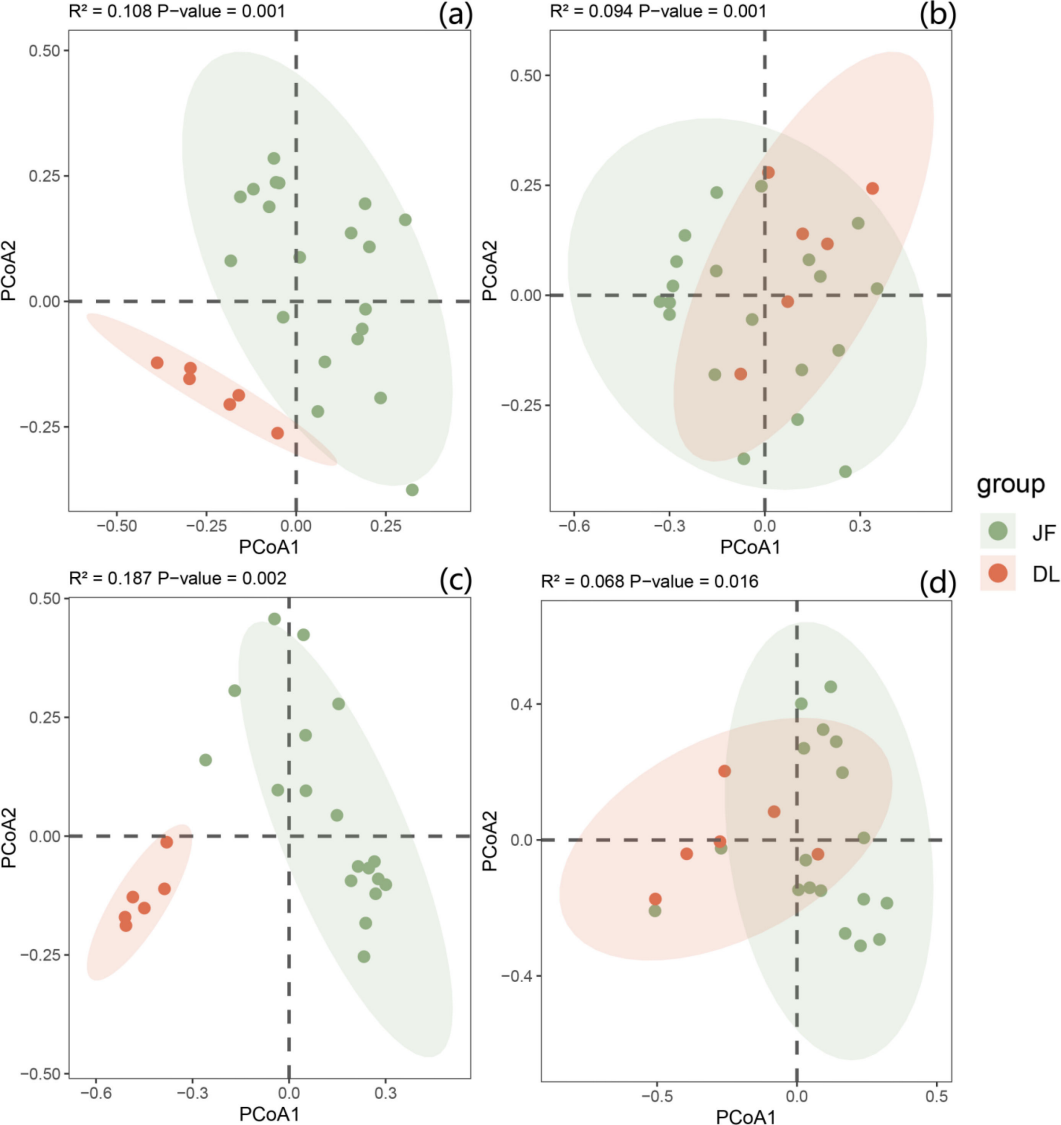
PCoA (principal co-ordinates analysis) of two communities jianfengling and diaoluoshan. **(a)** total layer, **(b)** arbor layer; **(c)** shrub layer; **(d)** herb layer; JF, Jianfengling area; DL, Diaoluoshan area.

Principal Coordinates Analysis (PCoA) was performed on the Chunia bucklandioides communities in the two distribution regions (**Figure 3**). The results revealed that the community structure of Chunia bucklandioides differed significantly between the two regions, both at the whole-community level and across different structural layers (all P < 0.05). Among these layers, the shrub layer had an R^2^ value of 0.187, with sampling plots showing a clear separation in the ordination space and the most significant difference in species composition. This indicated that the structural variation in the shrub layer was the key driver of the overall community structural differences between the two regions.

### 3.2 χ^2^ test analysis

As shown in **Table 3**, the variance ratio (VR) values (Equation 7) for all structural layers in communities containing *Chunia bucklandioides* exceed 1, which suggested a consistent positive overall interspecific association among the arbor, shrub and herb layers of the target community. In the Jianfengling area, the W-statistic (Equation 8) for both the arbor and shrub layers falls outside the range of χ^2^_0.95_(20) < W < χ^2^_0.05_(20), whereas the herb layer falls within this range, confirming significant overall interspecific associations in the arbor and shrub layers, but not in the herb layer. Similarly, in the Diaoluoshan area, the W-statistic for the arbor and shrub layers lies outside the range of χ^2^_0.95_(6) < W < χ^2^_0.05_(6), while the herb layer within from this range, further supporting significant overall interspecific associations in the arbor and shrub layers, but not in the herbaceous stratum.

**Table 3:** Overall connectivity of dominant species in the *Chunia bucklandioides* community table.

Distribution area	Forest layer	Variance (VR)	Statistic (W)	*χ*^2^threshold	Results
Jianfengling Area	Arbor layer	1.789	35.78	(10.85,31.41)	Significant positive association
	Shrub layer	2.107	42.14	(10.85,31.41)	Significant positive association
	Herb layer	1.338	26.76	(10.85,31.41)	Insignificant positive association
Diaoluoshan Area	Arbor layer	3.229	19.374	(1.64,12.59)	Significant positive association
	Shrub layer	3.655	21.99	(1.64,12.59)	Significant positive association
	Herb layer	1.438	8.628	(1.64,12.59)	Insignificant positive association

### 3.3 Overall association of different structural layers in communities containing *Chunia bucklandioides*

**Figure 4:**
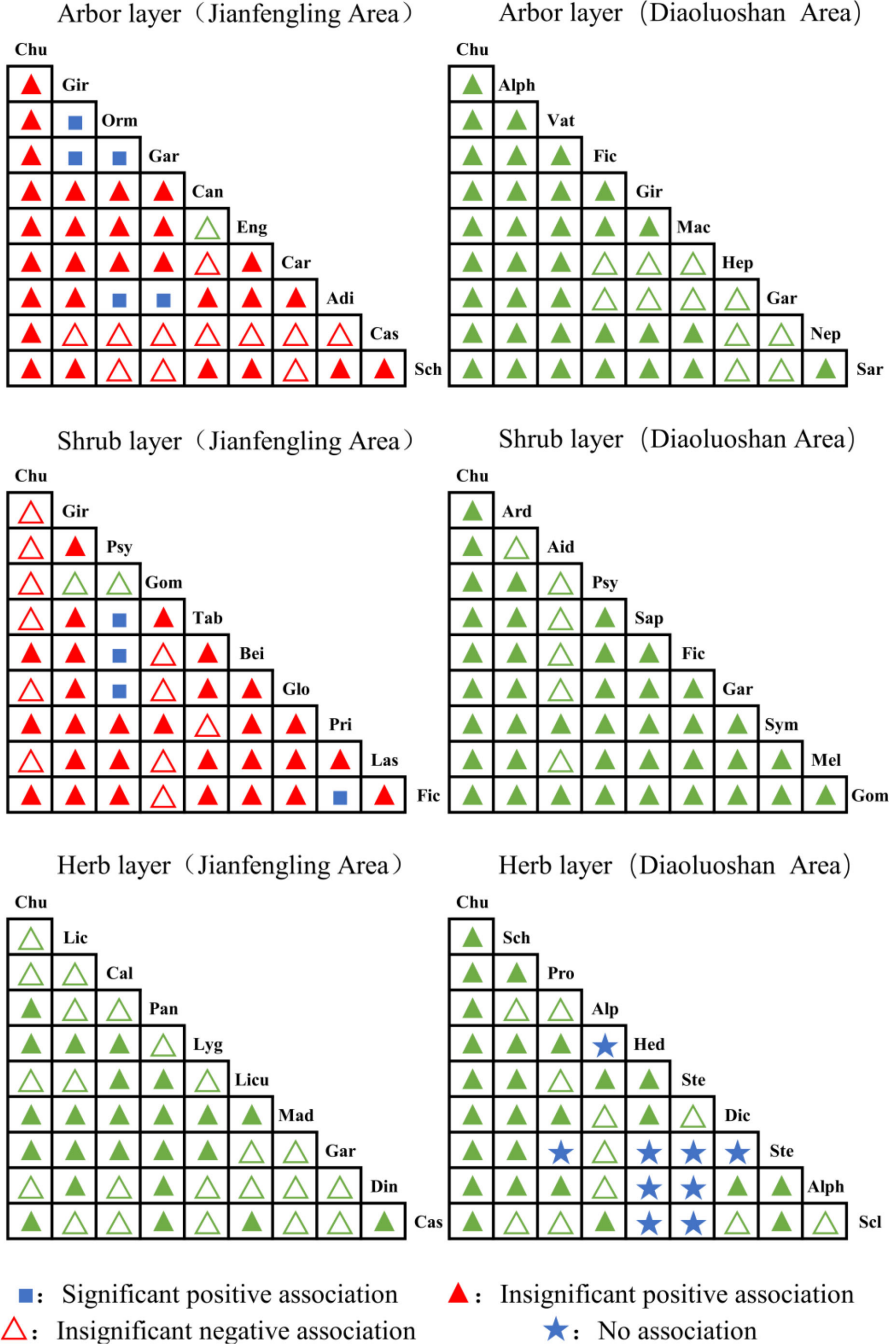
The *χ*^2^ semi-matrix diagram of interspecific association of dominant species in each forest layer. Species Abbreviation Reference **Table 2**.

The χ^2^ test (Equation 9) results for each forest layer across the two distribution regions are shown in **Figure 4**.

Arbor Layer: In the Jianfengling community, the ratio of positive to negative species pair associations was 2.75, with 5 pairs showing significant positive associations, while the remaining positive associations were non-significant. In contrast, the Diaoluoshan community had a positive-to-negative association ratio of 3.09, with all positive associations among species pairs being non-significant.

Shrub Layer: The ratio of positive to negative species pair associations in the Jianfengling community (2.46) was substantially lower than in the Diaoluoshan community (6.50), indicating fewer positive interspecific interactions in the former. Specifically, the Jianfengling community contained 4 species pairs with significant associations, including 6 species that showed negative associations with *Chunia bucklandioides*. In contrast, all interspecific interactions in the Diaoluoshan shrub layer were non-significant.

Approximately half of the species pairs in both regions exhibited non-significant positive interactions. The remaining species pairs showed distinct patterns: in the Jianfengling community, all other species pairs showed negative associations, with 4 species negatively affecting the growth of *Chunia bucklandioides*. In the Diaoluoshan community, however, 9 species pairs showed no association, and 11 species pairs showed negative associations—none of which involved *Chunia bucklandioides*, meaning these species posed no detrimental impact on its growth.

### 3.4 Association coefficient and percentage co-occurrence analyses

**Figure 5:**
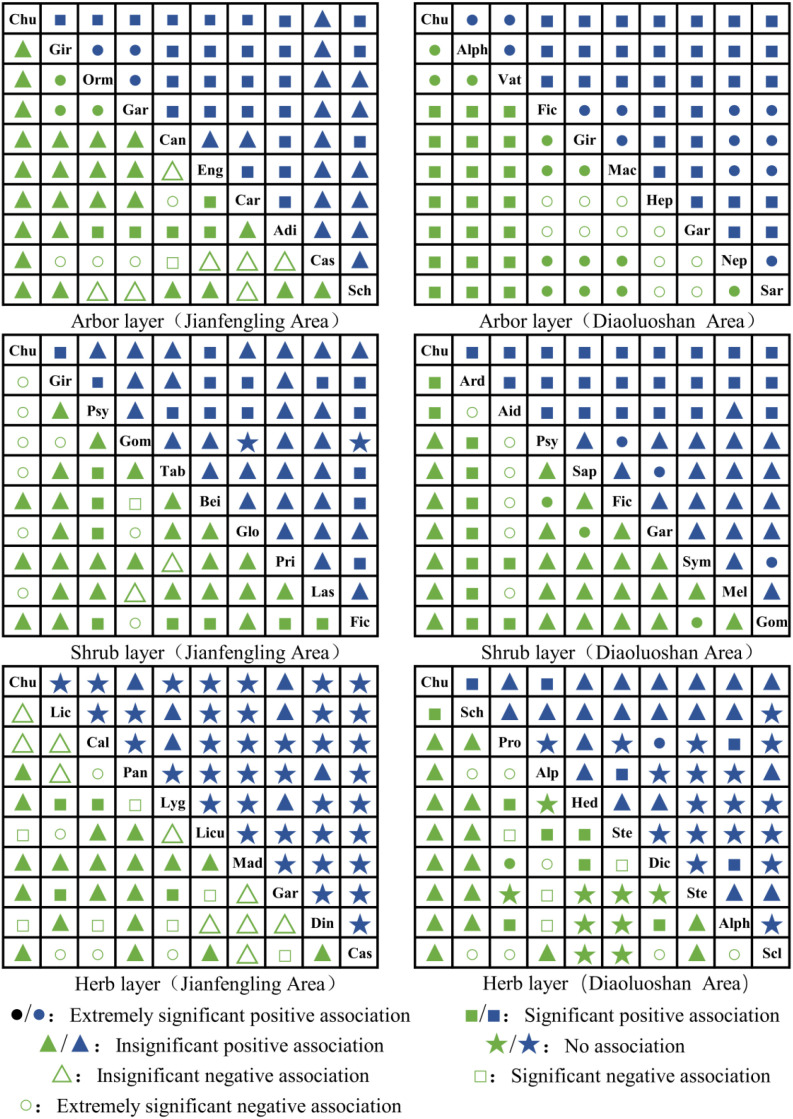
The semi-matrix diagram of dominant interspecific association coefficient *AC* and co-occurrence percentage *PC* in each forest layer of *Chunia bucklandioides* community. Note: 1) *AC* value range: 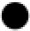
(Extremely significant positive association): *AC*<0.6; 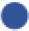
(Significant positive association): 0.3>*AC* ≤ 0.6; 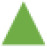
(Insignificant positive association): 0>*AC* ≤ 0.3; 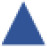
(No association): *AC* = 0; 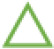
(Insignificant negative association): -0.3≤*AC* > 0; 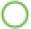
(Significant negative association): -0.6≤*AC*≤0.3; 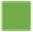
(Extremely significant negative association): *AC*>-0.6; 2) *PC* value range: 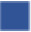
(Extremely significant positive association): *PC*<0.9; 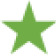
(Significant positive association): 0.6>*PC* ≤ 0.9; 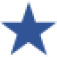
(Insignificant positive association): 0.3>*PC* ≤ 0.6; 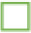
(No association): *PC* ≤ 0.3; 3) Species Abbreviation Reference **Table 2**.

The ratio of positive to negative interspecific associations revealed by the association coefficient (AC) (Equations 10–12) test was consistent with the results of the χ^2^ test, with the AC test further strengthening the interspecific associations (**Figure 5**).

Arbor Layer: In the Jianfengling community, 3 species pairs exhibited extremely significant positive associations, 5 showed significant positive associations, and 4 displayed extremely significant negative associations. In the Diaoluoshan community, all positive associations were further strengthened, with 13 species pairs showing extremely significant positive associations and 21 showing significant positive associations. Notably, the pairs *Chunia bucklandioides*–*Alphonsea monogyna* and *Chunia bucklandioides*–*Vatica mangachapoi* exhibited extremely significant positive associations.

Shrub Layer: In the Jianfengling community, the intensity of positive interspecific associations was lower than that of negative associations, with 10 species pairs showing extremely significant negative associations. Specifically, six dominant species—including *Gironniera subaequalis*, *Psychotria asiatica*, and *Gomphandra tetrandra*—exerted extremely significant adverse effects on the survival of *Chunia bucklandioides*. In the Diaoluoshan community, the interspecific association patterns were as follows: 3 species pairs exhibited extremely significant positive associations, 11 showed significant positive associations, 6 displayed extremely significant negative associations, and the remaining pairs had non-significant positive associations.

Herb Layer: In the Jianfengling community, the dominant species composition was complex, and interspecific interactions were predominantly negative, with all extremely significant associations being negative. In the Diaoluoshan community, 9 species pairs showed no association, 11 exhibited significant negative associations (including 7 pairs with extremely significant negative associations), and the majority of species pairs displayed positive associations.

The PC (Equation 13) results indicated that in the arbor and shrub layers of communities containing *Chunia bucklandioides*, associations were predominantly positive. Most of these associations were significant positive, with a smaller proportion showing extremely significant positive associations and non-significant positive associations. Only two species pairs in the Jianfengling community exhibited no association. In the herb layer, species pairs were mainly characterized by no association. The Jianfengling community had only 7 species pairs with non-significant positive associations, with all others showing no association. In the Diaoluoshan community, a total of 19 species pairs exhibited no association.

### 3.5 Community stability across different structural layers in Chunia bucklandioides-containing communities

**Figure 6:**
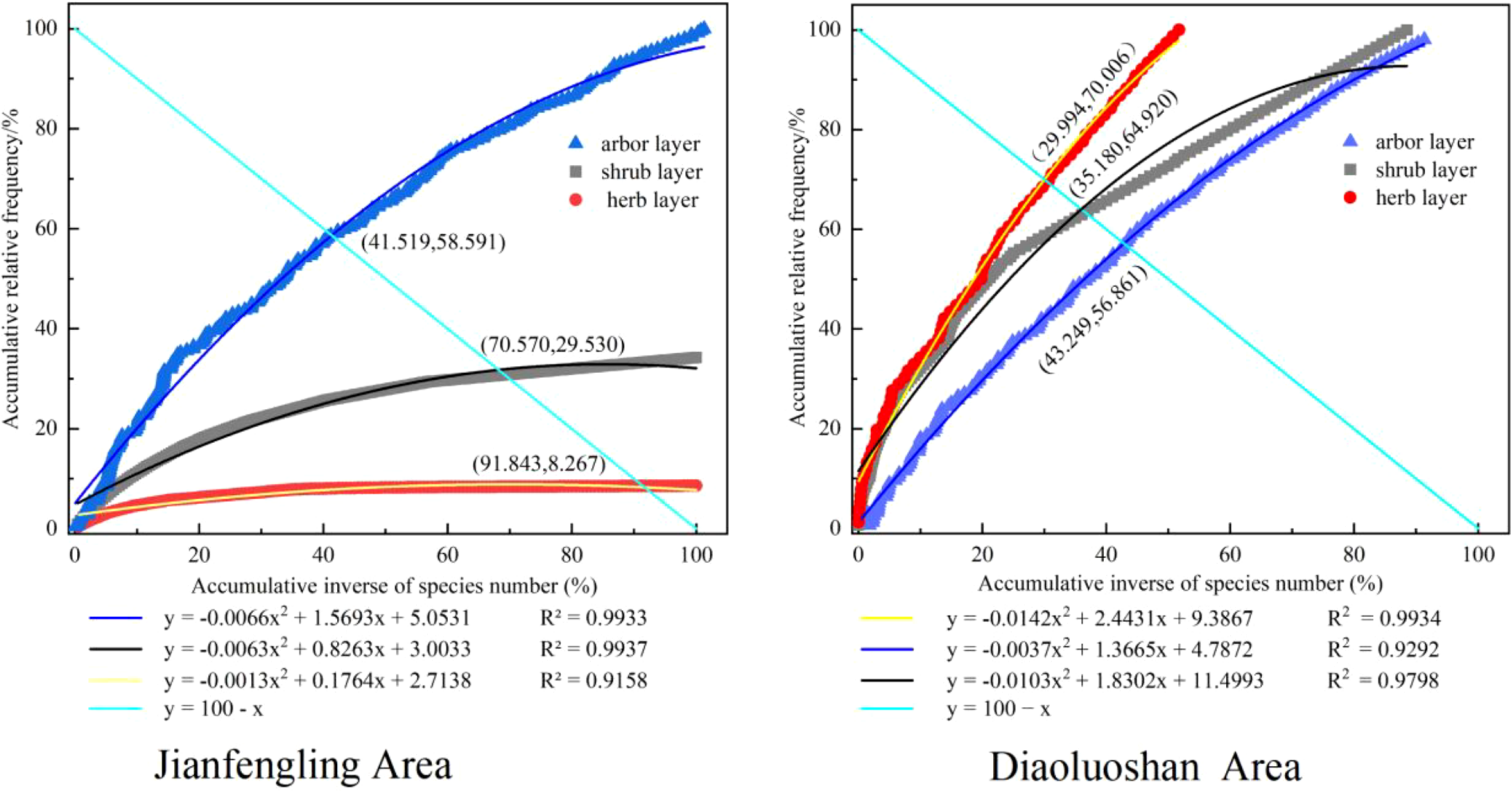
The stability and characterization of each forest layer in the communities where the *Chunia bucklandioides* of the two regions are located.

**Figure 6** shows the Euclidean distances from the intersection of the Godron stability fitting curve and the linear equation Y = 100 − X to the stability point (20, 80) for each structural layer in the two study regions (Equation 14). The distances are Jianfengling community (Arbor–shrub–herb): 30.397, 71.446, 101.506 and Diaoluoshan community (Arbor–shrub–herb): 32.801, 21.397, 14.204. Overall, the Euclidean distance values were relatively large across all layers in both regions, suggesting that the communities containing *Chunia bucklandioides* have not yet reached a stable state. The stability of the arbor layer was similar between the two regions, whereas the shrub and herb layers exhibited notable differences.

Regionally, the *Chunia bucklandioides* community in Diaoluoshan demonstrated greater stability than that in Jianfengling, particularly in the shrub and herb layers. Furthermore, the stability hierarchy differed between the two regions: Jianfengling showed an order of Arbor layer > Shrub layer > Herb layer, whereas Diaoluoshan exhibited an order of Herb layer > Shrub layer > Arbor layer.

## 4 Discussion

### 4.1 Species composition across different structural layers in *Chunia bucklandioides*-containing communities

The communities containing *Chunia bucklandioides* are primarily found in lowland rainforest ravine areas at mid-to-high elevations on Hainan Island. These areas are characterized by high soil moisture and rich species diversity. In the two study regions, a total of 354 plant species (within 0.8 ha) and 262 plant species (within 0.24 ha) were recorded in the *Chunia bucklandioides*-containing communities. This species richness is significantly higher than that found in other lowland rainforests, such as Los Tuxtlas in Mexico (234 species in a 1 ha plot) (Bongers et al., 1988) and Tamil Nadu in India (244 species in a 0.3 ha plot) (Swamy et al., 2000). The vegetation in these communities is dominated by angiosperms, with Lauraceae and Rubiaceae being the most common families. This floristic composition aligns with previous studies on lowland rainforests in Hainan Island (Shang et al., 2025, Han et al., 2019). Most *Chunia bucklandioides*-containing communities are secondary forests recovering from disturbances. Natural regeneration after disturbance represents a critical early stage in secondary forest development, during which pioneer tree species play a vital role in shaping the future composition and structure of the forest stand (Gloor et al., 2024). *Chunia bucklandioides*, with its strong sprouting ability (Zhang et al., 2024), occupies an absolute dominant position in natural regeneration and serves as a pioneer species in community restoration, resulting in its importance value being higher than that of other species across all forest layers.

The PCoA results revealed significant differences in community structure of *Chunia bucklandioides* between the two distribution regions, both at the whole-community level and across different structural layers. Arbor layer species are key consumers of both biotic and abiotic resources within the community. During the initial stages of community succession, they have a competitive advantage in capturing light resources and demonstrate strong reproductive potential (Kong et al., 2023). As a result, in both study regions, the top 10 species ranked by importance value consistently displayed high fruiting rates. Notably, there were distinct variations in species composition between the two regions, with only *Gironniera subaequalis* and *Garcinia oblongifolia* being shared as dominant species. The species assemblages in the shrub and herb layers showed even more pronounced differences. Communities in the Jianfengling area were predominantly composed of heliophilous (sun-loving) plants, whereas those in the Diaoluoshan region were dominated by sciophilous (shade-tolerant) species.

Two key factors may contribute to these disparities in dominant plant composition. First, significant climatic variations—particularly temperature and rainfall—characterized the two regions. Jianfengling, located in southwestern Hainan Island, experiences lower annual rainfall and a longer dry season. In contrast, Diaoluoshan, in southeastern Hainan Island, receives abundant rainfall year-round, maintaining a consistently humid microclimate (Qiu et al., 2004). Second, the *Chunia bucklandioides*-containing communities in the two regions have experienced different levels of early anthropogenic and natural disturbance. Field investigations revealed that the *Chunia bucklandioides* habitat in Jianfengling was severely impacted by historical deforestation and the recent expansion of tourist trails, which have significantly reduced canopy closure. This reduction in shading has allowed heliophilous shrub species like *Blastus cochinchinensis* and *Glochidion coccineum* to effectively utilize environmental resources. Conversely, the *Chunia bucklandioides* habitat in Diaoluoshan, located in steep terrain, was less affected by historical deforestation and has undergone favorable recovery, resulting in high canopy closure. The understory layers in this community are dominated by sciophilous species, including *Meliosma angustifolia*, *Symplocos ovatilobata*, and *Hedyotis paridifolia*.

### 4.2 Interspecific relationships at different structural layers of the *Chunia bucklandioides* community

The overall interspecific associations in the *Chunia bucklandioides*-containing communities exhibited significant positive associations in the arbor and shrub layers of both study regions, whereas non-significant positive associations were observed in the herb layer. Interspecific associations can reflect the trajectory of community succession and the degree of community stability (Ying et al., 2025). When interspecific relationships are predominantly negative or absent, the community is generally in the early stages of succession and has low stability. As succession progresses favorably, species within the community tend to form positive associations (including significant positive associations), which promote growth and drive the community toward a stable state defined by mutual benefit and independent coexistence (Tu et al., 2022, Zhou et al., 2000).

The significant positive associations observed in the woody plant community containing *Chunia bucklandioides* suggest that these communities are in the middle to late stages of succession. This finding aligns with the work of Huang et al. (Huang et al., 2025), who studied the interspecific associations of dominant tree species in Quercus communities in the Qinling Mountains of China. However, this phenomenon may also be linked to the exceptional regenerative ability of *Chunia bucklandioides*, which maintains high biomass levels, drives the successional process, and helps maintain relative stability among species (Chen et al., 2007).

The χ^2^ test results showed that the ratio of positive to negative associations among species pairs was greater than 0 in all layers of the *Chunia bucklandioides*-containing communities across both regions, indicating that interspecific relationships were dominated by positive interactions. Analyses using the AC and PC tests revealed high significance in the arbor layer of the *Chunia bucklandioides* community, suggesting that this layer is progressing toward a stable state. In contrast, the shrub and herb layers exhibited more unstable species composition, which can be attributed to the high sprouting rate of *Chunia bucklandioides* and the low survival rate of its juvenile individuals (Gui et al., 2024). These factors hinder the establishment of stable, resource-complementary, or mutually beneficial relationships, leading to highly significant negative correlations between some species pairs in these layers, while most positive correlations remained non-significant. This finding is consistent with the results of Chen et al. (Chen et al., 2024), who studied the interspecific associations of dominant woody plants in the Mulinzi National Nature Reserve. From a regional comparative perspective, the positive associations among species were stronger in the Diaoluoshan community than in the Jianfengling community. This may be attributed to the differences in the successional stages of the communities between the two distribution regions (Johnson et al., 2021). The community in Diaoluoshan is characterized by a steep slope, low anthropogenic disturbance, and high canopy closure, with shade-tolerant tree species such as *Alphonsea monogyna* and *Ardisia crenata* dominating all forest layers; this community is undergoing rapid succession, maintains a relatively stable structure, and exhibits predominantly positive interspecific associations. In contrast, the community in Jianfengling is situated in a relatively flat terrain, with most plots adjacent to logging trails, firebreaks, and water diversion channels, experiencing severe anthropogenic disturbance in the early stage and thus undergoing slow restoration. This community has a relatively low density of upper canopy tree species in the arbor layer, coupled with a complex turnover of understory species and unstable interspecific interactions. These conditions have intensified competition for environmental resources within the community, resulting in a slow successional process and relatively weak positive interspecific associations.

*Chunia bucklandioides* possesses an extremely strong sprouting ability and a well-developed root system. As a mid-to-upper canopy tree in the community, mature individuals can occupy dominant light resources through canopy spatial differentiation, making it the absolute dominant species of the community. This species exhibits an overall positive interspecific association with other co-occurring species, and such positive associations are stronger than those of other rare and endangered plant species including *Cephalotaxus hainanensis* and *Camellia luteoflora* (Li et al., 2024, Wang et al., 2023). These characteristics indicate that *Chunia bucklandioides* maintains a favorable growth status in the community. In the arbor layer of both regions, *Chunia bucklandioides* exhibited positive associations with other dominant species. Notably, it formed extremely significant positive associations with *Alphonsea monogyna* and *Vatica mangachapoi* in the Diaoluoshan community. In the shrub and herb layers, however, the sprouted saplings and seedlings of *Chunia bucklandioides* often overlap in niche with other species (Shang et al., 2025), intensifying competition for environmental resources. This has led to negative associations between *Chunia bucklandioides* and some dominant species—particularly in the shrub and herb layers of Jianfengling. In this area, saplings of *Gironniera subaequalis*, *Psychotria asiatica*, *Gomphandra tetrandra*, *Tabernaemontana bufalina*, *Glochidion coccineum*, and *Lasianthus trichophlebus* exerted adverse effects on the growth of *Chunia bucklandioides*, resulting in extremely significant negative associations. This further indicates that juvenile *Chunia bucklandioides* face substantial survival pressure, limiting population regeneration. Notably, however, the competitive pressure on juvenile individuals of *Chunia bucklandioides* may be a result of its own life-history strategy. In communities recovering from disturbance, tree species with strong sprouting ability can rapidly occupy ecological niches via sprouting. In the early stage, the mother tree transports water and other nutrients to sprouting individuals through its root system, while the sprouting individuals synthesize additional energy through photosynthesis, forming a resource complementary relationship between the two (Noutcheu et al., 2023). With the progression of community succession, however, sprouting individuals develop independent root systems and grow autonomously, which disrupts the resource complementary balance with the mother tree that ceases to supply nutrients to them. Consequently, these juvenile sprouting individuals struggle to compete for environmental resources with other rapidly colonizing heliophytic plants, leading to increased survival pressure (Bond and Midgley, 1995).

### 4.3 Stability analysis of different structural layers in *Chunia bucklandioides* communities

The Euclidean distances from the intersection points (between the Godron stability index curves and the linear equation Y = 100 - X) to the theoretical stability point (20, 80) were relatively large across all structural layers in both study regions, indicating poor overall stability of the *Chunia bucklandioides* communities in both areas. In dynamic communities, stability is influenced by multiple factors, including intraspecific and interspecific competition, environmental conditions, and anthropogenic disturbances, with human activities having a particularly significant impact on successional trajectories (Shang et al., 2025).Anthropogenic disturbances such as modern commercial logging, road construction, and tourism activities have exerted certain impacts on the restorative succession of Chunia bucklandioides communities in both regions. Variations in disturbance intensity have led to inconsistent rates of community succession (Turner et al., 1998), consequently resulting in divergent community stability between the two distribution areas. The Jianfengling area of Hainan Tropical Rainforest National Park features relatively flat terrain; high-intensity commercial logging in the last century (Xu, 2010) and the early initiation and high degree of tourism development (Chen et al., 2023, Zhu et al., 2023) have subjected the local Chunia bucklandioides communities to prolonged and intense disturbances. This has rendered their successional processes highly vulnerable to interference and their community stability relatively lower than that of the Chunia bucklandioides communities in the Diaoluoshan area, with this discrepancy being particularly pronounced in the shrub and herb layers. Heliophytic plants dominate in the early to middle stages of restorative succession in disturbed communities; as succession proceeds, dominant arbor species become established, understory canopy closure increases, and regenerating species gradually shift to shade-tolerant plants (Pilon et al., 2020, Wang et al., 2022). Analysis of understory species composition in Chunia bucklandioides communities across the two regions shows that the Diaoluoshan communities are dominated by shade-loving species such as Ardisia crenata, Aidia canthioides, and Pronephrium simplex, whereas the Jianfengling communities are dominated by heliophilous species including Psychotria henryi, Glochidion coccineum, and Calamus tetradactylus. This indirectly indicates that the current successional stage of Chunia bucklandioides communities in Jianfengling lags behind that in Diaoluoshan, with a corresponding lower level of community stability. Therefore, we recommend strengthening the *in-situ* conservation of endangered plant communities dominated by Chunia bucklandioides in the Jianfengling area.

## 5 Conclusions

1. The communities inhabited by *Chunia bucklandioides* have rich species composition, and there are significant differences in community structure between the Jianfengling area and the Diaoluoshan area.
2. In both distribution regions, positive interspecific associations dominate among species in different forest layers of the *Chunia bucklandioides* communities, with strong interspecific association in the arbor layer and weak interspecific association in the shrub layer and herb layer.
3. Different structural layers of the *Chunia bucklandioides* communities in both the Jianfengling area and the Diaoluoshan area are in an unstable state. In terms of stability, the performance of the two distribution regions is as follows: Diaoluoshan area community > Jianfengling area community; the order is reversed among different forest layers—for the Jianfengling area: arbor layer > shrub layer > herb layer; for the Diaoluoshan area: herb layer > shrub layer > arbor layer.
4. Mature individuals of *Chunia bucklandioides* occupy an absolute dominant position in the community, while juvenile individuals are under highly competitive pressure, leading to restricted population regeneration.

In summary, in the future conservation of *Chunia bucklandioides* populations, natural recovery should be the main approach; when necessary, appropriate human intervention should be implemented, including properly removing species that have antagonistic effects on juvenile *Chunia bucklandioides* individuals and thinning a portion of juvenile sprouting individuals of Chunia bucklandioides. This will alleviate the survival pressure on mother trees and seedlings, promote the development of community succession in a direction favorable to *Chunia bucklandioides* populations, enhance the stability of community structure, and thus facilitate the recovery and conservation of *Chunia bucklandioides* populations.

## Data Availability

The original contributions presented in the study are included in the article/supplementary material, further inquiries can be directed to the corresponding author/s.
